# A Case of Diabetes

**Published:** 1790

**Authors:** Philip Werner

**Affiliations:** Surgeon of the British Factory at Algiers


### LONDON MEDICAL JOURNAL.

### I. *A Case of Diabetes. By Mr.* Philip Werner, *Surgeon of the British Factory at Algiers.*

ON the 18th of December, 1788, I was consulted by Solomon Bullhare, a Jew of this place, aged twenty-two years, a tall and thin, but muscular, young man, who had been accustomed to fatiguing journies by land, and to the carrying heavy loads, but who had hitherto been always healthy and vigorous.

The account he gave me was, that about a month before he had perceived his urine to be of a white colour, and that it continued to be so for several days without his experiencing the least pain or difficulty in voiding it, and also without his being sensible of the least alteration in his appetite, sleep, or general health. That, however, when a few days had elapsed, he had found himself somewhat weaker, had become more thirsty than usual, and that his mouth and throat had felt dry and parched; that about this period also he perceived that he voided his urine urine more frequently, and that he did not perſpire as he had been accuſtomed to do; but ſo far was he from ſuſpecting the colour of his urine to be the effect of any particular diſeaſe, and ſo little, upon the whole, was he affected by it, that at the end of a fortnight, from the firſt appearance of the white urine, he undertook a journey of ten days on a mule; and that on the road he had ate, drank, and ſlept well, but was ſooner fatigued than on his former journies.

On the day I firſt ſaw him I found his pulſe quick and ſmall; his ſkin hot and dry; his tongue white and dry; his face rather pale; and he obſerved to me that he was become much thinner; he was coſtive, complained much of thirſt, of a dryneſs of his mouth and throat, and of a ſenſation which he compared to a burning heat extending from his ſtomach up to his throat. He voided his urine frequently, and to the amount of about ten pints in the ſpace of four and twenty hours, which was about twice the quantity of what he drank. I found it of a whitiſh colour, inclining to a ſhade of red, and of the conſiſtence of milk. It had a peculiar, though not diſagreeable, ſmell, but not at all like that of urine. To the the tafte it was fopy, and a little faltifh, adhering to the gum when tafted, but without the fmalleft perceptible degree of fweetnefs. He experienced no difficulty in voiding it, but complained much of a pain and weaknefs of his loins.

He ftill continued to follow his bufinefs as ufual, but obferved that he grew tired fooner than formerly.

As he was coftive, I began with giving him rhubarb and cream of tartar in dofes fufficient to produce three or four copious ftools. At bed time he was directed to put his feet into warm water, to take a fcruple of Dovar's powder, and to cover himfelf more than ufual during the night. At the fame time he was advifed to adhere to a light, but generous, diet, to drink fparingly of wine, to defift altogether from fpirituous liquors, (which he had been ufed, as I found, to drink in large quantities) to be a little more warmly cloathed than ufual, and to take gentle exercife.

He continued for fome days to repeat the Dovar's powder at bed time, and, befides this, took twice a day fmall dofes of tincture of cantharides. A mixture of lime water and milk was recommended to him for his common drink; drink; and proper dofes of rhubarb were given occafionally to procure ftools.

During the firft ten days of this treatment I obferved but little alteration in his complaint, except that on the days he took the rhubarb his urine appeared to be thinner, clearer, and lefs in quantity. His fkin, however, ftill remained hot and dry, with only now and then a flight moifture on it at night. I now directed him to have recourfe to the warm bath every two or three days, and, laying afide the tincture of cantharides, gave him a fcruple of Dovar's powder, and the fame quantity of rhubarb, at firft twice, and afterwards three times a day. The ufe of the lime water and milk was ftill continued. Under this courfe his fkin gradually became moifter, and his ftools more regular; his urine at the fame time gradually leffened in quantity, became clearer and thinner, and began to have the natural urinous fmell again; and at the end of about five weeks he found himfelf well.

The following experiments were made on this patient's urine at the time he firft came under my care:

### EXPERIMENT I.

Some concentrated vitriolic acid being added to ſome of the urine in a glaſs veſſel, fell to the bottom, and remained there unmixed; but upon ſhaking the veſſel the urine gradually acquired a ſomewhat darker colour, and emitted a ſtrong ſmell like that of rotten eggs. The next morning a ſmall quantity of a cream-like ſubſtance was found on the ſurface of the urine, which was now of a light brown colour. The vitriolic acid, which was before white and clear, was become brown, and was ſtill at the bottom of the veſſel unmixed, ſo that I could bear to taſte the mixture without hurting my tongue.

### EXPERIMENT II.

Salt of tartar added to the urine gradually turned it to a yellowiſh colour; but no farther change was obſerved to take place in the mixture till the next morning, when it was found to emit a ſtrong urinous ſmell.

### EXPERIMENT III.

To another portion of the ſame urine ſome ſpirit of ſal ammoniac was added. This turned ed it to a more milk white colour; but no other alteration was produced.

### Experiment IV.

A pound of the urine was evaporated, by a gentle heat, to dryneſs. During the evaporation no urinous ſmell was perceptible, nor was there any oilineſs to be ſeen on its ſurface; but the liquor gradually acquired a brown colour. The extract obtained by this proceſs weighed ten drachms and a half, and was of the colour and confiſtence of brown ſugar, but taſted very ſalt.

### Experiment V.

Upon pouring ſome of the concentrated acid of vitriol on a portion of this extract, a ſtrong effervefcence enſued, and many fumes aroſe which had the pungent ſmell of the marine acid.

### Experiment VI.

Similar phænomena, but with more fumes and a ſtill ſtronger ſmell of the marine acid, took place upon pouring concentrated nitrous acid on the extract.

### Experiment VII.

On the addition of concentrated marine acid, no effervefcence nor any other change happened.

### Experiment VIII.

On the addition of lixivium tartari, a few very fmall air bubbles rofe to the furface for fome little time, and then gradually emitted a urinous fmell.

### Experiment IX.

Upon pouring fpirit of fal ammoniac on the extract, no fuch emiffion of air bubbles nor any other change was obferved to take place.

### Experiment X.

I put fome of the urine into a wide-mouthed vial, and covering it only flightly with a bit of paper, placed it on a fhelf where I might obferve its changes from day to day; but during the fpace of nearly two months I could perceive no change in it, excepting that a thin cream-like fubftance gradually appeared on its furface. After that time it acquired, by degrees, a darker colour; but four months elapfed before it became putrid.

